# Natural killer-cell immunoglobulin-like receptors trigger differences in immune response to SARS-CoV-2 infection

**DOI:** 10.1371/journal.pone.0255608

**Published:** 2021-08-05

**Authors:** Roberto Littera, Luchino Chessa, Silvia Deidda, Goffredo Angioni, Marcello Campagna, Sara Lai, Maurizio Melis, Selene Cipri, Davide Firinu, Simonetta Santus, Alberto Lai, Rita Porcella, Stefania Rassu, Federico Meloni, Daniele Schirru, William Cordeddu, Marta Anna Kowalik, Paola Ragatzu, Monica Vacca, Federica Cannas, Francesco Alba, Mauro Giovanni Carta, Stefano Del Giacco, Angelo Restivo, Simona Deidda, Antonella Palimodde, Paola Congera, Roberto Perra, Germano Orrù, Francesco Pes, Martina Loi, Claudia Murru, Enrico Urru, Simona Onali, Ferdinando Coghe, Sabrina Giglio, Andrea Perra

**Affiliations:** 1 Complex Structure of Medical Genetics, R. Binaghi Hospital, Local Public Health and Social Care Unit (ASSL) of Cagliari, Sardinian Regional Company for the Protection of Health (ATS Sardegna), Cagliari, Italy; 2 Association for the Advancement of Research on Transplantation O.d.V., Non Profit Organisation, Cagliari, Italy; 3 Department of Medical Sciences and Public Health, University of Cagliari, Cagliari, Italy; 4 Liver Unit, Department of Internal Medicine, University Hospital of Cagliari, Cagliari, Italy; 5 Complex Structure of Pneumology, SS Trinità Hospital, ASSL Cagliari, ATS Sardegna, Cagliari, Italy; 6 Complex Structure of Infectious Diseases, SS Trinità Hospital, ASSL Cagliari, ATS Sardegna, Cagliari, Italy; 7 Local Crisis Unit (UCL), ATS Sardegna, Cagliari, Italy; 8 Unit of Oncology and Molecular Pathology, Department of Biomedical Sciences, University of Cagliari, Cagliari, Italy; 9 Medical Genetics, Department of Medical Sciences and Public Health, University of Cagliari, Cagliari, Italy; 10 Colorectal Surgery Unit, Department of Surgical Science, University of Cagliari, Cagliari, Italy; 11 Molecular Biology Service Laboratory, Department of Surgical Science, University of Cagliari, Cagliari, Italy; 12 Clinical Chemical and Microbiology Laboratory, University Hospital of Cagliari, Cagliari, Italy; University of California San Francisco, UNITED STATES

## Abstract

**Background:**

The diversity in the clinical course of COVID-19 has been related to differences in innate and adaptative immune response mechanisms. Natural killer (NK) lymphocytes are critical protagonists of human host defense against viral infections. It would seem that reduced circulating levels of these cells have an impact on COVID-19 progression and severity. Their activity is strongly regulated by killer-cell immuno-globulin-like receptors (KIRs) expressed on the NK cell surface. The present study’s focus was to investigate the impact of KIRs and their HLA Class I ligands on SARS-CoV-2 infection.

**Methods:**

*KIR* gene frequencies, *KIR* haplotypes, KIR ligands and combinations of KIRs and their HLA Class I ligands were investigated in 396 Sardinian patients with SARS-CoV-2 infection. Comparisons were made between 2 groups of patients divided according to disease severity: 240 patients were symptomatic or paucisymptomatic (Group A), 156 hospitalized patients had severe disease (Group S). The immunogenetic characteristics of patients were also compared to a population group of 400 individuals from the same geographical areas.

**Results:**

Substantial differences were obtained for *KIR* genes, *KIR* haplotypes and *KIR*-*HLA* ligand combinations when comparing patients of Group S to those of Group A. Patients in Group S had a statistically significant higher frequency of the *KIR* A/A haplotype compared to patients in Group A [34.6% vs 23.8%, OR = 1.7 (95% CI 1.1–2.6); P = 0.02, Pc = 0.04]. Moreover, the *KIR2DS2*/*HLA C1* combination was poorly represented in the group of patients with severe symptoms compared to those of the asymptomatic-paucisymptomatic group [33.3% vs 50.0%, OR = 0.5 (95% CI 0.3–0.8), P = 0.001, Pc = 0.002]. Multivariate analysis confirmed that, regardless of the sex and age of the patients, the latter genetic variable correlated with a less severe disease course [OR_M_ = 0.4 (95% CI 0.3–0.7), *P*_*M*_ = 0.0005, *P*_*MC*_ = 0.005].

**Conclusions:**

The *KIR2DS2*/*HLA C1* functional unit resulted to have a strong protective effect against the adverse outcomes of COVID-19. Combined to other well known factors such as advanced age, male sex and concomitant autoimmune diseases, this marker could prove to be highly informative of the disease course and thus enable the timely intervention needed to reduce the mortality associated with the severe forms of SARS-CoV-2 infection. However, larger studies in other populations as well as experimental functional studies will be needed to confirm our findings and further pursue the effect of KIR receptors on NK cell immune-mediated response to SARS-Cov-2 infection.

## Introduction

Since the emergence of COVID-19 which was formally declared a pandemic in March 2020 by the World Health Organization [1], a large range of pharmaceutical and non-pharmaceutical measures have been adopted in the hope to counter the effects of infection with the SARS-CoV-2 virus but, so far, none of these have led to radical changes in the management of patients [2]. The recent availability of safe and effective vaccines represents a major breakthrough but it may take months or even years in some countries before the effects of the pandemic can be efficiently mitigated. Meanwhile repeated lockdowns and other extreme preventive strategies have somewhat succeeded in curbing the spread of the virus.

The large majority of infected individuals are asymptomatic, pre-symptomatic or present with mild flu-like symptoms such as low grade fever and/or a dry cough. Unfortunately, many of these individuals remain undetected (~50%) and unknowingly continue to spread the virus [3]. In mild cases, the virus mainly affects the upper respiratory tract, particularly the large airways [4]. Unfortunately, some individuals (20%) become seriously ill and often require hospitalisation for ventilatory support. It has been established that 4–6% of infected individuals develop critical and life-threatening symptoms.

Respiratory complications ranging from pneumonia to acute respiratory distress syndrome (ARDS) are the hallmark of severe COVID-19. However, the clinical course may also be constellated by neurological and gastrointestinal manifestations, heart failure, renal failure, liver damage, hypercoagulability, septic shock and multi-organ failure (MOF). Elderly male patients with comorbidities are those at the highest risk of a severe prognosis [5–9]. Moreover, alarming news continues to mount on both young and elderly patients–commonly referred to as “long haulers”—who have recovered from the disease but continue to have symptoms. Several hypotheses have been put forward to offer a plausible explanation for long-term lingering COVID-19 symptoms but none have managed to solve the problem. It is suspected that the immune systems of such patients may continue to overreact or perhaps trigger lasting changes in immune response mechanisms long after the infection has passed and patients have stopped shedding the virus.

Never before have so many world scientists and researchers focused on a single disease with such urgency that within less than a year, the scientific community has acquired a deep knowledge of the structure and functioning of the SARS-CoV-2 virus, its modes of transmission and the unusually variegated clinical pictures [10, 11]. The clinical features of infected individuals do not soley depend upon viral load [12]. As previously observed in the SARS and MERS epidemics, the coordinated activities of host innate and adaptive immunity are critical to effective control of infection, viral clearance and evolution of the disease [13–16]. Combined innate and adaptive responses of the host, confronted with the virulence and capacity of the virus to evade these host responses, will eventually dictate outcome of the disease [16].

Like in other respiratory RNA viral infections, human airway epithelial cells are among the first targets to encounter SAR-CoV-2. These cells represent a first line of defense and have a major role in triggering inflammatory response through specific receptors called pattern recognition receptors (PRRs) [17]. Toll-like receptor 3 (TLR3), TLR7, TLR8, melanoma differentiation-associated protein 5 (MDA-5) and retinoic acid-inducible gene I (RIG-I) are PRRs expressed by immune and non-immune cells that are critical to recognition of viruses by the innate immune system, especially RNA viruses such as those of the coronaviridae family [16]. Sensing through PRRs leads to the transcription of genes involved in the inflammatory response, with the production of type I interferons (IFNs), particularly IFN-a/b, being an essential step in antiviral response [18]. Type I IFNs are produced by many immune and non-immune cells [16, 19, 20] and elicit intrinsic antiviral responses [21]. In particular, they are essential to prime innate and adaptive lymphocytes, including natural killer (NK) cells [22].

Natural Killer cells are fundamental components of the innate and adaptative immune systems [23] and have an important role in the response to viral infections in both humans and animal models [24–26]. Despite their beneficial antiviral activity, NK cells have been implicated as mediators of immunopathology in infectious diseases, such as those caused by respiratory syncytial virus (RSV) [27], influenza A virus [28–32], hepatitis B virus [33] and hepatitis C virus [34].

Some authors have suggested that NK cells may also play an important role in SARS-CoV-1 infection. They found that the total number of NK cells and the percentage of CD158b+ NK cells (NK cells KIR2DL2/3+ and KIR2DS2+) were significantly lower in patients with SARS-CoV-1 infection than in healthy subjects. Moreover, NK cell expression of CD158b+ correlated with disease severity and the presence of anti-SARS coronavirus–specific antibodies [35].

NK cells only represent 5 to 10% of peripheral blood circulating lymphocytes but are a prominent lymphocyte population in human and mouse lungs. In the healthy human lung, NK cells make up for 10 to 20% of all lymphocytes, most of which are CD56dimCD16+. NK cells in the lung represent a highly differentiated population with a CD57+NKG2A− phenotype and high KIR expression [36]. This phenotipe (CD57+KIRs+ and the absence of the inhibitory receptor NKG2A) characterizes the so-called memory-like NK cells which seem to have a decisive role in countering SARS-CoV-2 [37, 38]. In view of these considerations, it is plausible to hypothesize that KIR receptors play an important role in modulating the immune response of NK cells against SARS-CoV-2 infection.

NK cells rely on the balance of both inhibitory and activating receptor signals to determine which target cells will be attacked or tolerated. Killer immunoglobulin-like receptors (KIRs) expressed on the NK cell surface are considered to be protagonists among the receptors regulating NK cell responsiveness. The *KIR* locus comprises two broad groups of haplotypes based on gene content. Group A haplotypes contain the *KIR3DL3*, *KIR2DL1*, *KIR2DL3*, *KIR2DL4*, *KIR3DL1*, and *KIR3DL2* inhibitory *KIR* genes along with *KIR2DS4* as the only activating *KIR* gene whereas Group B haplotypes have a variable gene content with one or more genes encoding activating KIRs (*KIR2DS1*, *KIR2DS2*, *KIR2DS3*, *KIR2DS5* and *KIR3DS1)* and genes encoding inhibitory KIRs (*KIR2DL2*, *KIR2DL5A* and *KIR2DL5B)* [39–41].

KIR receptors expressed on the NK cell surface regulate their functions through binding to human leukocyte antigen (HLA) Class I ligands on cells. HLA-C is the predominant ligand for KIR on NK cells. KIRs recognize two groups of HLA-C allotypes based on a single amino acid substitution at position 80 of the alpha-1 domain of the alpha helix. HLA-C group 1 alleles (*HLA C1*) have an asparagine at this position whereas HLA-C group 2 alleles (*HLA C2*) have a lysine [42, 43].

The *KIR2DL2* and *KIR2DL3* inhibitory receptors and the *KIR2DS2* activating receptor bind molecules of the C1 group while the *KIR2DL1* inhibitory receptor and the *KIR2DS1* activating receptor bind molecules of the C2 group. Additionally, it has been shown that the *KIR3DL1* inhibitory receptor and also the *KIR3DS1* activating receptor can interact with *HLA-B* molecules expressing the Bw4 epitope. The binding strength of these KIR/HLA ligands is determined by the presence of an amino acid residue at position 80 of the Bw4 molecule (Bw4Ile80 are stronger ligands for their specific receptors, KIR3DS1 and KIR3DL1, than Bw4Thr80) [44]. However, variations in KIR-ligand interactions and binding affinities may further complicate this scenario. A typical example is that some alleles of *KIR2DL2* and *KIR2DL3* also interact with specific HLA antigens of the C2 group [45].

Sardinia with its relatively high level of genetic homogeneity is among the Italian regions with the lowest rates of SARS-CoV-2 infection and mortality. Thousands of years of isolation are responsible for the unique genetic characteristics of the Sardinian population which, in the case of COVID-19, may possibly represent an advantage. This background is particularly useful when we need to understand which defense mechanisms of innate and adaptive immunity are deployed in the host to combat viral infection.

The present study of the Sardinian population was aimed at investigating the impact of NK cells on the development and course of COVID-19 with particular emphasis on the *KIR* gene repertoire and the dual function exerted by HLA class I molecules as preferential ligands for NK cell KIR receptors and CD8+ T cell activators.

## Materials and methods

A panel of 396 patients were recruited from 1 June to 1 December 2020. The diagnosis of SARS-CoV-2 infection was confirmed in all patients by RT-PCR from nasopharyngeal swab. The patients were assigned to one of two groups according to disease severity: 156 patients with severe disease were assigned to Group S and 240 patients who were either asymptomatic or paucisymptomatic (a-paucisymptomatic) were assigned to Group A. Patients in Group A had been confined to home isolation whereas patients in Group S had been hospitalized in the Covid Unit of the S.S.Trinità Hospital in Cagliari. Fourteen of the 156 hospitalized patients died from cardio-respiratory arrest related to severe pulmonary impairment (interstitial pneumonia).

The large majority of patients in Group S required high-flow nasal oxygen supplementation or invasive treatment with mechanical ventilation. The second group of patients (Group A) were a- paucisymptomatic with symptoms such as a runny nose, loss of taste or smell, headaches, a dry cough and/or other flu-like symptoms [46].

A panel of 400 individuals of Sardinian origin going back at least two generations were selected from the regional bone marrow donor registry to represent the genetic background of the Sardinian population [47]. This population group appropriately represented the male-to-female ratio and genetic profiles of the population in the central-south geographical areas from where the COVID-19 patients were recruited.

The *KIR* genes, *KIR* haplotypes and, *KIR*-*HLA* ligand combinations observed in the 396 patients with SARS-CoV-2 infection were compared to those of the 400 unrelated individuals within the population group. Analogous analyses were performed to compare the two groups of patients (Group S vs Group A). Comparisons of the KIR ligand frequencies (*HLA-A*, *-B*, *-C* allele groups) were also performed.

### Ethics statement

Patients were recruited and enrolled in the study protocol at the Department of Medical Sciences and Public Health of the University of Cagliari, the University Hospital of Cagliari (AOUCA) and the SS.Trinità Hospital of the Sardinian Regional Company for the Protection of Health (ATS Sardegna). Written informed consent was obtained from all patients and controls in accordance with the ethical standards (institutional and national) of the local human research committee. The study protocol, including informed consent procedures, conforms to the ethical guidelines of the Declaration of Helsinki and was approved by the responsible ethics committee (Ethics Committee of the Cagliari University Hospital; date of approval: May, 27, 2020; protocol number GT/2020/10894). Records of written informed consent are kept on file and are included in the clinical record of each patient.

### *HLA* and *KIR* gene typing

DNA from nasopharyngeal swab was extracted with the Qiagen QIAamp DNA Mini Kit (Qiagen, Valencia, CA, USA) according to the manufacturer’s instructions. Briefly, each swab was washed with PBS (200 μl). The recovered washing buffer was mixed with 20 μl of proteinase k and 200 μl of the AL buffer was added to the mix. After 10 minutes of incubation at 56°C, the sample was processed according to the kit instructions. Purity and quantification of total DNA were assessed with the NanoDrop 1000 Spectrophotometer (Thermo Fisher Scientific).

Patients and the population group were typed for *HLA* alleles using a next generation sequencing (NGS) platform. The samples were genotyped for Class I (*HLA-A*, *-B*, and *-C*), and Class II (*HLA-DRB1*, *HLA-DQA1*, *HLA-DQB1* and *HLA-DPB1*) loci using a commercially available NGS 7‐Loci amplification kit (Omixon Holotype HLA^TM^, for MiSeq Illumina®), according to the manufacturer’s instructions. *HLA* genotypes were assigned using HLA Twin software (Omixon, Inc).

Rare or ambiguous *HLA* alleles were methodically re-typed according to the Sanger sequencing-based typing (SBT) method using the following SBT kits: AlleleSEQR®HLA for the *HLA-A*, *-B*, *-C* and *-DRB1* loci and SBTexcellerator® for the *HLA-DQA1*, *-DQB1* and *-DPB1* loci (GenDx & GenDx Products, Utrecht, The Netherlands).

*HLA-C* alleles were assigned to the *C1* or *C2* ligand group according to the presence of asparagine or lysine at position 80 of the *HLA-C* molecule. *HLA-B* alleles were classified as Bw4 or Bw6 depending on the amino acids between positions 77 and 83.

The two isoforms of *HLA-Bw4* were distinguished by the presence of leucine (Bw4Ile80*)* or threonine (Bw4Thr80) in position 80. *HLA-A23*, *-A24*, and *-A32* pertain to the HLA-Bw4Ile80 group of serological epitopes [44].

Genomic DNA from both patients and the population group was typed for the presence of the 14 *KIR* genes *KIR2DL1*, *KIR2DL2*, *KIR2DL3*, *KIR2DL4*, *KIR2DL5*, *KIR3DL1*, *KIR3DL2*, *KIR3DL3*, *KIR2DS1*, *KIR2DS2*, *KIR2DS3*, *KIR2DS4*, *KIR2DS5* and *KIR3DS1* using PCR-SSP with primers specific for each locus according to a previously reported method [48–50].

KIR typing was validated on a panel of 192 randomly selected samples taken from patients and individuals of the population group by PCR-SSP using a commercial kit (Olerup SSP® KIR Genotyping– https://labproducts.caredx.com/products/olerup-ssp/kir/?ProductNo=104.101-12&LotNo=9L3). The two methods yielded 99.8% concordance for the presence or absence of the *KIR* genes.

*KIR2DS4* alleles with a 22 base pair (bp) deletion variant in exon 5 (*KIR2DS4* alleles *003, *004, *006, *010, *012 and *013) were distinguished from functional, full-length *KIR2DS4* alleles (*KIR2DS4* FL) using the method and set of primers described by Yawata et al. [51]. This made it possible for us to establish which patients only carried the deletion variants (DV) of *KIR2DS4* (*KIR2DS4* DV) and, therefore, completely lacked NK cells expressing functional activating *KIRs*.

### Haplotype group assignment and *KIR*-ligand combinations

*KIR* haplotypes were assigned as previously described [48, 52, 53] with group B haplotypes defined by one or more of the following genes: *KIR2DL5*, *KIR2DS1*, *KIR2DS2*, *KIR2DS3*, *KIR2DS5* and *KIR3DS1* and Group A haplotypes characterized by the absence of all these genes [54].

Patients were stratified into two groups according to homozygosity for *KIR* A haplotype (*KIR* haplotype AA), heterozygosity or homozygosity for *KIR* B haplotype. *KIR* haplotype AA was compared to *KIR* haplotype B/x (AB and BB groups combined) [5, 55].

All *KIR* haplotypes contain a combination of four centromeric (*cA01*, *cB01*, *cB02*, *cB03*) and two telomeric (*tA01*, *tB01*) gene-content motifs. The *cB03* motif is present on a single haplotype, whereas the remaining three centromeric and two telomeric motifs can be present on all *KIR* haplotypes. The centromeric and telomeric regions of the *KIR* haplotypes in this study were assigned as previously described [56, 57]. Analyses were based on the content of the inhibitory (L-long) or activating (S-short) *KIR* genes and their frequencies among the population group and COVID-19 patients with different clinical manifestations. Particular focus was put on KIR-HLA ligand interactions because of their essential role in the educational process of functional and potentially alloreactive NK cell clones.

### Statistical analysis

First of all, we analyzed a series of clinical and demographic characteristics and genetic traits in the two groups of patients stratified for being a-paucisymptomatic (Group A) or hospitalized with severe symptoms and/or clinical manifestations of SARS-CoV-2 infection (Group S).

Subsequently, we compared the *KIR* gene frequencies, *KIR* haplotypes, *KIR* ligands and combinations of *KIRs* and their *HLA* Class I ligands between the entire group of 396 patients and the population group of 400 individuals. And finally, the aforesaid genetic variables were also compared between Group S and Group A COVID-19 patients.

We computed mean and standard deviation (SD) for all continuous variables and used percentages and 95% confidence intervals (95% CI) for categorical data. The two-tailed Fisher’s exact test was used to compute all P values and calculate the odds ratios (OR) with a 95% CI for categorical data. The Student test was used for all continuous variables. Only P values below 0.05 were considered to be statistically significant.

Considering the large size of the data sets of *KIR* genes, *HLA* ligands and clinical conditions used for association analysis, all significant P values were adjusted by the conservative Bonferroni correction method for multiple comparisons. In particular, a corrective factor of 11 (14 *KIR* genes minus the 3 framework *KIR* genes present in all subjects) was used to calculate the Pc values for differences in *KIR* gene frequencies observed between the different groups.

All calculations and statistical analysis for evaluation of the *KIR* haplotypes in patients and the population group were performed using a specific programming code written with R language (© 2016 The R Foundation–R Core Team 2020) version 4.0.3 [58].

Multivariate logistic regression analysis was performed to calculate the independence from age and gender of the clinical and genetic variables that were included in the comparisons between the two groups of patients and whose P values yielded statistically significant results in the univariate analysis (P < 0.05). Given that at least 10 observations per variable is crucial for obtaining valid and reliable results with the logistic regression model, the beta-thalassemic trait and the three-loci *HLA* haplotype (HLA-B*58:*01*, C*07:*01*, DRB1*03:*01*) were excluded from the analysis. Age and gender were the most relevant factors and therefore the P value and odds ratios of the ten statistically significant factors were adjusted accordingly. The multivariate P values (P_M_) were corrected for multiple comparisons by the Bonferroni method (P_MC_).

**Fig 1:**
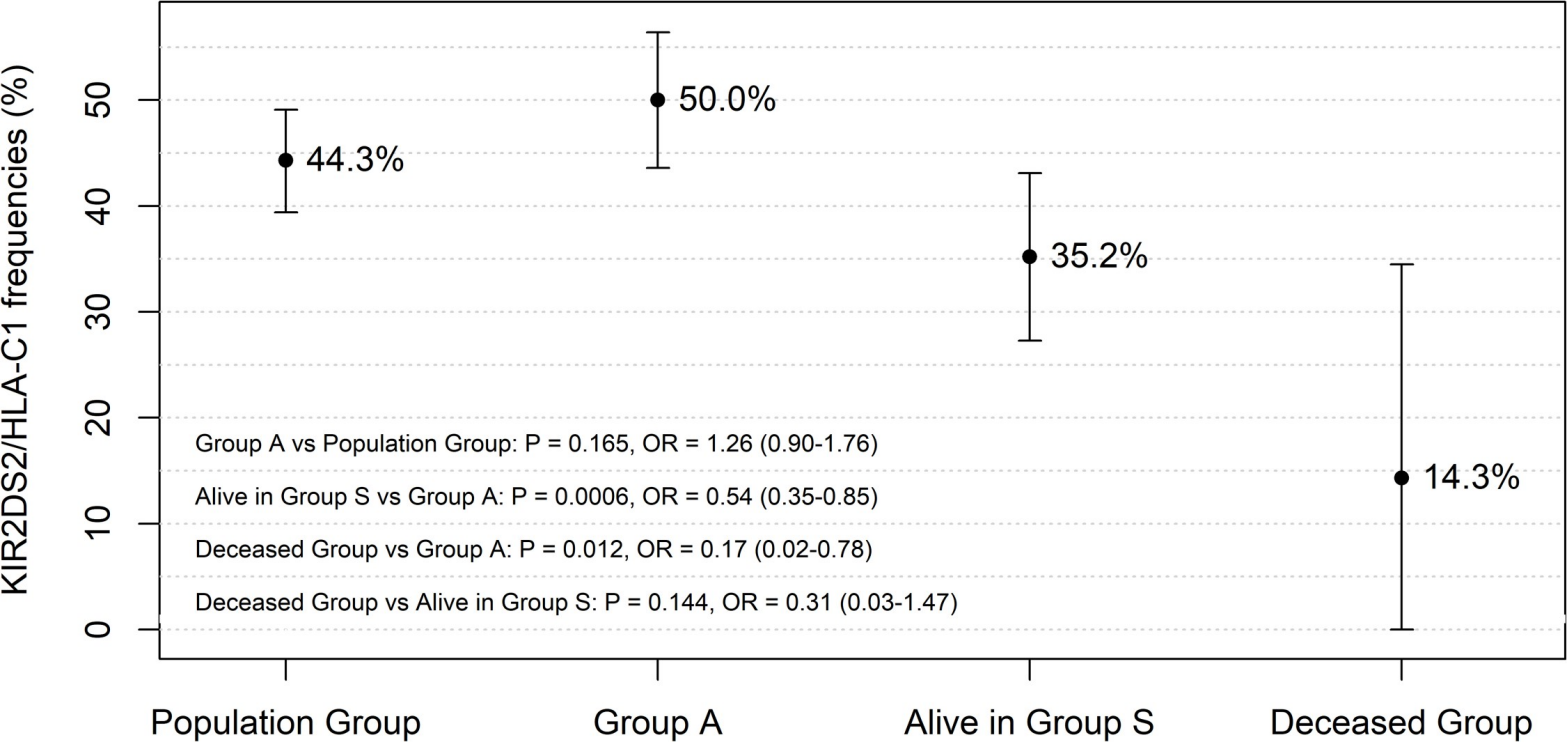
Frequency of the *KIR2DS2*/*HLA C1* functional unit compared between patients stratified according to the severity of COVID-19 clinical manifestations [Group A (N = 120/240, 50.0%), alive in Group S (N = 50/142, 35.2%) and deceased patients in group S (N = 2/14, 14.3%)]. The comparison between Group A (N = 120/240, 50.0%) and the population group (N = 177/400, 44.3%) showed a similar frequency in the two groups. The error bars represent the 95% confidence intervals of the *KIR2DS2*/*HLA C1* frequency in each group of patients and the population group.

From the analyses, it emerged that regardless of age and sex, the functional unit *KIR2DS2*/*HLA C1* was the most informative genetic predictor of less severe outcomes in patients with SARS-CoV-2 infection. This prompted us to evaluate the frequency of the *KIR2DS2*/*HLA C1* functional unit in the population group (N = 177/400), Group A patients (N = 120/240), alive patients in Group S (N = 50/142) and deceased patients in Group S (N = 2/14). The comparisons are illustrated in Fig 1, where the error bars represent the 95% confidence intervals of the *KIR2DS2*/*HLA C1* frequency in each of the considered groups.

## Results

### Main clinical and genetic characteristics of Sardinian COVID-19 patients

Table 1 shows the main clinical and genetic features of SARS-CoV-2 positive patients. Mean age at diagnosis was 55.9 years (mean ± SD: 55.9 ± 17.5; 95% CI 49.7–56.4). More specifically, 36.4% (n = 144) of the patients had an age of up to 50 years; 30.3% (n = 120) of the patients were over 65 years of age. Also in our study, adults over 65 years experienced more severe symptoms and clinical manifestations [OR = 3.4 (95% CI 2.1–5.5), P = 5.5∙10^−8^]. The incidence of COVID-19 was more or less the same in males and females with only a slightly higher percentage of female patients (50.8%). However, female patients would seem to be less vulnerable to the severe effects of COVID-19. Indeed, in our study 111 of the 156 critically ill patients were males [71.2% vs 28.8%, OR = 4.6 (95% CI 2.9–7.3), P = 1.6∙10^−12^].

**Table 1:** Comparisons of baseline clinical, genetic and biochemical parameters between COVID-19 patients with severe disease (Group S) and a-paucisymptomatic patients (Group A).

Characteristics of Sardinian COVID-19 patients	Total patients (N = 396)		Group A (N = 240)		Group S (N = 156)		Comparison Group S vs Group A	
	Mean ± SD	95% CI	Mean ± SD	95% CI	Mean ± SD	95% CI	P value	Δx (95% CI)^9^
Age (yr)	55.9 ± 17.5	49.7–56.4	50.8 ± 17.4	46.5–52.4	63.7 ± 14.7	60.8–72.1	2.2·10^−13^	12.9 (12.3–13.5)
	**n (%)**	**95% CI**	**n (%)**	**95% CI**	**n (%)**	**95% CI**	**P value**	**OR (95% CI)**
Age ≤ 50 yr	144 (36.4)	31.6–41.1	117 (48.8)	42.4–55.1	27 (17.3)	11.3–23.3	9.0·10^−11^	0.2 (0.1–0.4)
50 yr < Age < 65 yr	132 (33.3)	28.7–38.0	75 (31.3)	25.4–37.1	57 (36.5)	28.9–44.2	0.278	1.3 (0.8–2.0)
Age ≥ 65 yr	120 (30.3)	25.8–34.8	48 (20.0)	14.9–25.1	72 (46.2)	38.3–54.0	5.5·10^−8^	3.4 (2.1–5.5)
Male	195 (49.2)	44.3–54.2	84 (35.0)	28.9–41.1	111 (71.2)	64.0–78.3	1.6·10^−12^	4.6 (2.9–7.3)
Female	201 (50.8)	45.8–55.7	156 (65.0)	58.9–71.1	45 (28.8)	21.7–36.0	1.6·10^−12^	0.2 (0.1–0.3)
FLU vaccine 2019	52 (13.1)	9.8–16.5	39 (16.3)	11.6–20.9	13 (8.3)	4.0–12.7	0.023	0.5 (0.2–0.9)
***Comorbidities***								
Cancer	7 (1.8)	0.5–3.1	6 (2.5)	0.5–4.5	1 (0.6)	0.0–1.9	0.252	0.3 (0.0–2.1)
Diabetes	14 (3.5)	1.7–5.4	6 (2.5)	0.5–4.5	8 (5.1)	1.6–8.6	0.176	2.1 (0.6–7.5)
Chronic pulmonary disease^1^	4 (1.0)	0.0–2.0	4 (1.7)	0.0–3.3	0	0	0.157	
Ischemic heart disease^2^	29 (7.3)	4.7–9.9	21 (8.7)	5.2–12.3	8 (5.1)	1.6–8.6	0.236	0.6 (0.2–1.4)
Hypertension	58 (14.6)	11.2–18.1	35 (14.6)	10.1–19.1	23 (14.7)	9.1–20.4	1	1.0 (0.5–1.9)
Autoimmune disease^3^	75 (18.9)	15.1–22.8	33 (13.7)	9.4–18.1	42 (26.9)	19.9–33.9	0.002	2.3 (1.3–4.0)
Hypercholesterolemia	41 (10.4)	8.0–14.2	19 (7.9)	4.5–11.3	22 (14.1)	10.2–21.8	0.063	1.9 (1.0–3.7)
***Chronic Medication use***								
Steroidal anti-inflammatory drug	21 (5.3)	3.1–7.5	9 (3.8)	1.3–6.2	12 (7.7)	3.5–11.9	0.108	2.1 (0.8–5.9)
Non steroidal anti-inflammatory drug^4^	23 (5.8)	3.5–8.1	12 (3.8)	2.2–7.8	11 (7.1)	3.0–11.1	0.390	1.4 (0.6–3.7)
ACE II inhibitor^5^	41 (10.4)	7.3–13.4	21 (8.8)	5.2–12.3	20 (12.8)	7.5–18.1	0.237	1.5 (0.8–3.1)
Angiotensin II receptor blocker^6^	22 (5.5)	3.3–7.8	12 (5.0)	2.2–7.8	10 (6.4)	2.5–10.3	0.654	1.3 (0.5–3.4)
Beta and calcium channel blockers^7^	54 (13.6)	10.2–17.0	27 (11.3)	7.2–15.3	27 (17.3)	11.3–23.3	0.100	1.6 (0.9–3.0)
Levothyroxine	24 (6.1)	3.7–8.4	19 (7.9)	5.2–12.3	5 (3.2)	0.0–4.1	0.083	0.4 (0.1–1.1)
***Genetic trait***								
Beta-thalassemic Trait	30 (7.8)	5.0–10.2	30 (12.5)	8.3–16.7	0	0	1.8·10^−7^	
G6PDH deficiency	45 (11.4)	8.2–14.5	24 (10.0)	6.2–13.8	21 (13.5)	8.1–18.9	0.332	1.4 (0.7–2.7)
HLA-B*58:*01*, C*07:*01*, DRB1*03:*01*^8^	12 (3.0)	1.5–4.9	12 (5.0)	2.2–7.8	0	0	0.004	
***Serology***	**Mean ± SD**	**95% CI (IQR)**	**Mean ± SD**	**95% CI (IQR)**	**Mean ± SD**	**95% CI (IQR)**	**P value**	**Δx (95% CI)**^9^
White blood cell count (x10^3^/μL)	8.2 ± 2.8	7.4–8.9 (4.5)	8.1 ± 2.3	7.7–8.4 (4.5)	8.4 ± 3.7	7.6–9.3 (4.6)	0.352	0.3 (0.0–1.1)
Lymphocyte count (x10^3^/μL)	1.1 ± 0.6	1.0–1.3 (0.5)	1.2 ± 0.5	1.1–1.2 (0.7)	1.0 ± 0.8	0.8–1.2 (0.6)	0.112	0.1 (0.0–0.3)

^1^ Chronic obstructive pulmonary disease was defined as a diagnosis of emphysema and/or bronchitis.

^2^ Ischemic heart disease was categorized as history of myocardial infarction or angina.

^3^Autoimmune diseases included 20 cases of Hashimoto’s thyroiditis, 12 cases of type I diabetes mellitus, 13 cases of rheumatoid arthritis, 9 cases of autoimmune hepatitis and 21 cases of other autoimmune disorders.

^4^ Non-steroidal anti-inflammatory drugs included aspirin, ibuprofen, diclofenac, naproxen, indomethacin, celecoxib, and meloxicam.

^5^ Angiotensin converting enzyme inhibitors included captopril, enalapril, lisinopril, fosinopril, ramipril, and quinapril.

^6^ Angiotensin II receptor blockers included losartan, candesartan, irbesartan, olmesartan, and valsartan.

^7^ Dihydropyridine calcium channel blockers included amlodipine, nifedipine. Beta blockers included atenolol, bisoprolol, labetalol, metoprolol, nebivolol.

^8^None of the patients with SARS-CoV-2 infection in Group S, carried the extended haplotype HLA-A*02:*05*, B*58:*01*, C*07:*01*, DRB1*03:*01* which was present in 5.3% of the 400 individuals of the population group selected for being highly representative of the Sardinian population [OR = 0 (95% CI 0–0.5), P = 0.002].

^9^ Mean difference (for continuous variables): Δx = |x_1_ (patients in Group S)—x_2_ (patients in Group A)|.

Abbreviations: SD = standard deviation; CI = confidence interval; IQR = interquartile range.

Another parameter that could possibly have an influence on the COVID-19 disease course is the seasonal flu vaccine [59]. We found a lower frequency of vaccinated subjects in the hospitalized patients (Group S) compared to the a-paucisymptomatic patients isolated at home (Group A) [8.3% vs 16.3%, OR = 0.5 (95% CI 0.2–0.9), P = 0.023]. However, the small sample size of vaccinated patients (13%) yielded a confidence interval that was too wide to draw conclusions.

Alongside sex and age, several comorbidities have been implicated in the disease course of COVID-19. However, evidence for the association of comorbidities to the risk of severe and fatal outcomes is still incomplete. In our cohort of patients, 7.3% presented ischemic heart disease, 14.6% arterial hypertension, 10.4% hypercholesterolemia, 18.9% autoimmune diseases and 3.5% type I diabetes mellitus. To investigate the possibility of association, we compared Group S and Group A patients for each comorbidity present in the total cohort of patients. Hypercholesterolemia was more common in Group S, but did not reach statistical significance. [14.1% vs 7.9%, OR = 1.9 (95% CI 1.0–3.7), P = 0.063]. The only significant difference found for comorbidities was a higher frequency of autoimmune disorders in the group of patients with severe disease [26.9% vs 13.7%, OR = 2.3 (95% CI 1.3–4.0), P = 0.002]. These were Hashimoto’s thyroiditis (12 in Group S vs 8 in Group A), type I diabetes mellitus (8 in Group S vs 4 in Group A), rheumatoid arthritis (9 in Group S vs 4 in Group A), autoimmune hepatitis (6 in Group S vs 3 in group A), and other diseases with an autoimmune etiopathogenesis (7 in group S vs 14 in Group A).

Taken separately, none of these autoimmune disorders were significantly associated with a more severe disease course. Even diabetes, which is widely reported to be associated with increased COVID-19 mortality, did not reach statistical significance in our study.

Chronic drug intake was similar among the two groups of patients. No significant differences were found for G6PDH enzyme deficiency. In line with our previous study [59], none of the severely ill patients carried the β^0^39-thalassemia mutation of the beta globin chain [0% vs 12.5%, P = 1.8∙10^−7^]. Moreover, the three-loci *HLA* haplotype HLA-B*58:*01*, C*07:*01*, DRB1*03:*01* was completely absent in the 156 patients of Group S [0% vs 5.0%, P = 0.004] which confirms a possible protective effect of this haplotype in the Sardinian population.

### Differences in *KIR* genes and *KIR* haplotype frequencies between the population group and COVID-19 patients

Table 2 shows the differences between 396 Sardinian COVID-19 patients and 400 individuals of the population group for frequencies of activating and inhibitory *KIR* genes, *KIR* haplotypes and *KIR* gene motifs.

**Table 2:** *KIR* genes and genotype frequencies compared between patients and the population group.

	Population group (400)	COVID-19 patients (396)	P value	OR (95% CI)	Pc*
	n (%)	n (%)			
**Inhibitory *KIR* genes**					
***2DL1***	381 (0.953)	393 (0.992)	0.001	6.5 (1.9–34.7)	0.011
***2DL2***	232 (0.580)	249 (0.629)	0.17	1.2 (0.9–1.6)	1
***2DL3***	343 (0.858)	372 (0.939)	0.0002	2.6 (1.5–4.4)	0.0022
***2DL4***	400 (1.00)	396 (1.00)			
***2DL5***	223 (0.558)	231 (0.583)	0.48	1.1 (0.8–1.5)	1
***3DL1***	373 (0.933)	369 (0.932)	1	1.0 (0.5–1.8)	1
***3DL2***	400 (1.00)	396 (1.00)			
***3DL3***	400 (1.00)	396 (1.00)			
**Activating *KIR* genes**					
***2DS1***	155 (0.388)	175 (0.442)	0.13	1.3 (0.9–1.7)	1
***2DS2***	231 (0.578)	244 (0.616)	0.28	1.2 (0.9–1.6)	1
***2DS3***	145 (0.363)	147 (0.371)	0.82	1.0 (0.8–1.4)	1
***2DS4***	376 (0.940)	378 (0.955)	0.43	1.3 (0.7–2.7)	1
***- 2DS4 DV+***	334 (0.835)	324 (0.818)	0.58	0.9 (0.6–1.3)	1
**- *2DS4 FL+***	125 (0.313)	123 (0.311)	1	1.0 (0.7–1.4)	1
***2DS5***	127 (0.318)	144 (0.364)	0.18	1.2 (0.9–1.7)	1
***3DS1***	148 (0.370)	165 (0.417)	0.19	1.2 (0.9–1.6)	1
***Genotypes***					
***A/A***	115 (0.288)	111 (0.280)	0.87	0.97 (0.7–1.3)	1
**- *2DS4 DV/DV***	72 (0.180)	69 (0.174)	0.85	0.96 (0.7–1.4)	1
**- *2DS4 DV/FL and FL/FL***	43 (0.108)	42 (0.106)	1	0.99 (0.6–1.5)	1
***B/x***	285 (0.713)	285 (0.720)	0.87	1.0 (0.8–1.4)	1
***Centromeric KIR* motifs**					
***cA01/cA01***	169 (0.423)	144 (0.364)	0.09	0.8 (0.6–1.0)	1
***cA01/cB01*, *cA01/cB02***	176 (0.440)	228 (0.575)	0.0001	1.7 (1.3–2.3)	0.0003
***cB01/cB01*, *cB01/cB02*, *cB02/cB02***	55 (0.138)	24 (0.061)	0.0003	0.4 (0.2–0.7)	0.0009
***Telomeric KIR* motifs**					
***tA01/tA01***	237 (0.592)	207 (0.523)	0.05	0.7 (0.6–1.0)	0.16
***tA01/tB01***	139 (0.348)	156 (0.394)	0.19	1.2 (0.9–1.6)	0.56
***tB01/tB01***	24 (0.060)	33 (0.083)	0.22	1.4 (0.8–2.6)	0.65

*P values were calculated for comparisons between Sardinian COVID-19 patients and the population group. Pc corresponds to P values corrected for multiple comparisons.

Abbreviations: OR = odds ratio; CI = confidence interval; B/x = *KIR* haplotypes AB and BB; *2DS4 DV+ =* deletion variant alleles of the *KIR2DS4* gene; *2DS4 DV/DV* ***=*** homozygous for deletion variant alleles of the *KIR2DS4* gene; *2DS4 DV/FL and FL/FL* = heterozygous and homozygous for full-length allele variants of the *KIR2DS4* gene. Group A and B *KIR* haplotypes have distinctive centromeric and telomeric *KIR* gene-content motifs as described by Pyo CW et al. [56]. No *cB03* motifs were found in COVID-19 patients or the population group.

Overall, the frequency of *KIR* genes with an inhibitory role on NK cell function was increased in patients compared to the population group. In particular, statistically significant increments were observed for the *KIR2DL1* and *KIR2DL3* inhibitory *KIR* genes in patients [99.2% vs 95.3%, OR = 6.5 (95% CI 1.9–34.7), P = 0.001: Pc = 0.011 and 93.9% vs 85.8%, OR = 2.6 (95% CI 1.5–4.4), P = 0.0002, Pc = 0.0022, respectively]. No significant differences were observed between the two groups for the number of activating *KIR* genes. Homozygosity for *KIR* A haplotype displayed a similar distribution in the two groups of patients and the population group and could therefore be excluded as having a potential role in disease susceptibility.

Analysis of the centromeric regions of the *KIR* locus revealed a higher frequency of the *cA01/cB01*, *cA01/cB02 KIR* haplotype motifs (characterized by the *KIR2DL3*, *KIR2DS2* and/or *KIR2DL2* genes) in patients with SARS-CoV-2 infection compared to the population group [57.6% vs 44.0%, OR = 1.7 (95% CI 1.3–2.3), P = 0.0001, Pc = 0.0003]. Consequently, *cA01/cA01* and *cB01/cB01*, *cB01/cB02*, *cB02/cB02 KIR* haplotype motifs had a reduced frequency in patients [36.4% vs 42.3%, OR = 0.8 (95% CI 0.6–1.0), P = 0.38 and 6.1% vs 13.8%, OR = 0.4 (95% CI 0.2–0.7), P = 0.0003, Pc = 0.0009, respectively]. No significant differences were observed in comparisons of the telomeric region of the KIR locus between patients and the population group.

Furthermore, all genes characterizing the centromeric and telomeric regions of the *KIR* locus were analyzed in combination with their respective *HLA* ligands (*KIR*-*HLA* functional units). The results are reported in Table 3.

**Table 3:** Comparisons of *KIR* genes and their cognate *HLA* ligands between COVID-19 patients and the population group.

	Population group (400)	COVID-19 patients (396)	P value	OR (95% CI)
	n (%)	n (%)		
***KIR* Ligands**				
***C1/C1***	109 (0.273)	102 (0.258)	0.69	0.9 (0.7–1.3)
***C2/C2***	98 (0.245)	111 (0.280)	0.26	1.2 (0.9–1.7)
***C1/C2***	193 (0.483)	183 (0.462)	0.57	0.9 (0.7–1.2)
***HLA Bw4***	301 (0.753)	306 (0.773)	0.51	1.1 (0.8–1.6)
**- *Bw4* Ile80**	269 (0.673)	267 (0.674)	1.00	1.0 (0.7–1.4)
**-*Bw4* Thr80**	77 (0.193)	93 (0.235)	0.17	1.3 (0.9–1.8)
***HLA Bw6***	344 (0.860)	336 (0.848)	0.69	0.9 (0.6–1.4)
**Activating *KIR/HLA* ligands**				
***2DS1+*/*HLA-C2*+**	119 (0.298)	139 (0.351)	0.11	1.3 (0.9–1.7)
***2DS2+*/*HLA-C1*+**	177 (0.443)	172 (0.434)	0.83	1.0 (0.7–1.3)
*2DS4+*/HLA-A*11+ or -C*04+	105 (0.263)	101 (0.255)	0.87	1.0 (0.7–1.3)
***3DS1+*/*HLA-Bw4*+**	118 (0.295)	123 (0.311)	0.64	1.1 (0.8–1.5)
**Inhibitory *KIR/HLA* ligands**				
***2DL1+*/*HLA C2*+**	278 (0.695)	294 (0.742)	0.16	1.3 (0.9–1.7)
***2DL2+*/*HLA C1+***	178 (0.445)	177 (0.447)	1.00	1.0 (0.8–1.3)
***2DL3+*/*HLA C1+***	259 (0.648)	267 (0.674)	0.45	1.1 (0.8–1.5)
***3DL1+*/*HLA Bw4+***	282 (0.705)	288 (0.727)	0.53	1.1 (0.8–1.5)
***2DL2-3+/HLA C1+***	137 (0.343)	159 (0.402)	0.09	1.3 (1.0–1.7)

The diverse combinations of activating and inhibitory *KIR* genes with their cognate HLA ligands were analyzed and compared between the population group and patients.

Abbreviations: OR = odds ratio; CI = confidence interval; + = present.

A substantial overlap of *HLA* ligand (*HLA C1* group, *HLA C2* group and Bw epitopes) group frequencies was observed in patients and the population group. Overall, patients had more inhibitory *KIR*-*HLA* ligand combinations than those exerting an activating function.

In fact, all inhibitory functional units examined were found to have higher frequencies in COVID-19 patients than in the population group (*KIR2DL1*/*HLA C2* group 74.2% vs 69.5%, *KIR2DL2*/*HLA C1* group 44.7% vs 44.5%, *KIR2DL3*/*HLA C1* group 67.4% vs 64.8%, *KIR3DL1*/*HLA* Bw4 epitope 72.7% vs 70.5% and *KIR2DL2-3*/*HLA C1* group 40.2% vs 34.3%). However, none of these combinations achieved statistical significance, including the combination represented by *KIR2DL2*-3/*HLA C1* [40.2% vs 34.3%, OR = 1.3 (95% CI 1.0–1.7) P = 0.09].

### Correlation of *KIR* genes and haplotype frequencies to the clinical manifestations of SARS-CoV-2 infection

The results of the analysis of *KIR* genes and haplotypes in patients with SARS-CoV-2 infection appeared to be very interesting in relation to the severity of the clinical picture (Table 4).

**Table 4:** *KIR* genes and genotype frequencies compared between patients divided according to clinical manifestations.

	Group A 240 patients	Group S 156 patients	P value*	OR (95% CI) A vs S	Pc^
	n (%)	n (%)			
**Inhibitory *KIR* genes**					
***2DL1***	237 (0.987)	156 (1.00)	0.28		1
***2DL2***	162 (0.675)	87 (0.558)	0.02	0.6 (0.4–0.9)	0.22
***2DL3***	222 (0.925)	150 (0.962)	0.19	2.0 (0.8–5.2)	1
***2DL4***	240 (1.00)	156 (1.00)			
***2DL5***	147 (0.613)	84 (0.538)	0.15	0.7 (0.5–1.1)	1
***3DL1***	222 (0.925)	147 (0.942)	0.54	1.3 (0.6–3.0)	1
***3DL2***	240 (1.00)	156 (1.00)			
***3DL3***	240 (1.00)	156 (1.00)			
**Activating *KIR* genes**					
***2DS1***	114 (0.475)	61 (0.391)	0.12	0.7 (0.5–1.1)	1
***2DS2***	162 (0.675)	82 (0.526)	0.003	0.5 (0.3–0.8)	0.033
***2DS3***	79 (0.329)	68 (0.436)	0.07	1.5 (0.9–2.3)	0.77
***2DS4***	231 (0.962)	147 (0.942)	0.46	1.6 (0.6–4.0)	1
***- 2DS4 DV+***	195 (0.812)	129 (0.827)	0.79	1.1 (0.7–1.9)	1
**- *2DS4 FL+***	69 (0.287)	54 (0.346)	0.22	1.3 (0.9–2.0)	1
***2DS5***	96 (0.400)	48 (0.308)	0.07	0.7 (0.4–1.0)	0.77
***3DS1***	102 (0.425)	63 (0.404)	0.75	0.9 (0.6–1.4)	1
***Genotypes***					
***A/A***	57 (0.238)	54 (0.346)	0.02	1.7 (1.1–2.6)	0.04
**- *2DS4 DV/DV***	36 (0.150)	33 (0.202)	0.14	1.5 (0.9–2.6)	0.28
**- *2DS4 DV/FL and FL/FL***	21 (0.088)	21 (0.135)	0.18	1.6 (0.9–3.1)	0.36
***B/x***	183 (0.762)	102 (0.654)	0.02	0.6 (0.4–0.9)	0.04
***Centromeric KIR* motifs**					
***cA01/cA01***	75 (0.313)	69 (0.442)	0.01	1.7 (1.1–2.7)	0.03
***cA01/cB01*, *cA01/cB02***	147 (0.612)	81 (0.519)	0.08	0.7 (0.5–1.0)	0.24
***cB01/cB01*, *cB01/cB02*, *cB02/cB02***	18 (0.075)	6 (0.039)	0.19	0.5 (0.2–1.3)	0.57
***Telomeric KIR* motifs**					
***tA01/tA01***	123 (0.513)	84 (0.538)	0.68	1.1 (0.7–1.7)	1
***tA01/tB01***	96 (0.400)	60 (0.385)	0.83	0.9 (0.6–1.4)	1
***tB01/tB01***	21 (0.087)	12 (0.077)	0.85	0.9 (0.4–1.8)	1

*P values were calculated for comparisons between patients with severe clinical manifestations (Group S) and a-paucisymptomatic patients (Group A).

^Pc represents the P values corrected for multiple comparisons. Abbreviations: OR = odds ratio; CI = confidence interval; B/x = *KIR* haplotypes AB and BB; *2DS4 DV+ =* deletion variant alleles of the *KIR2DS4* gene; *2DS4 DV/DV* ***=*** homozygous for deletion variant alleles of the *KIR2DS4* gene; *2DS4 DV/FL and FL/FL* = heterozygous and homozygous for full-length allelic variants of the *KIR2DS4* gene. Group A and B *KIR* haplotypes have distinctive centromeric and telomeric *KIR* gene-content motifs as described by Pyo CW et al. [56].

The most interesting finding emerging from the analysis of the *KIR* genes and genotype frequencies evaluated in the two groups of patients divided according to the severity of the clinical manifestations was represented by the significant reduction observed for the *KIR2DS2* activating *KIR* gene and the *KIR2DL2* inhibitory *KIR* gene in Group S compared to group A [52.6% vs 67.5%, OR = 0.5 (95% CI 0.3–0.8), P = 0.003, Pc = 0.033 and 55.8% vs 67.5%, OR = 0.6 (95% CI 0.4–0.9), P = 0.02, Pc = 0.22 respectively].

However, only *KIR2DS2* maintained statistical significance after correction for multiple comparisons, thus suggesting a protective effect of this gene against the severe clinical manifestations of SARS-CoV-2 infection.

In our population group, like in other Caucasian populations, the *KIR2DS2* and *KIR2DL2* genes are found in strong linkage disequilibrium [60].

It is interesting to note that in our cohort of patients with SARS-CoV-2 infection, particularly those with severe symptoms (Group S), we observed different frequencies for these two genes. In fact, five patients of Group S had *KIR* gene profiles characterized by the presence of *KIR2DL2* and the absence of *KIR2DS2*. Although such *KIR* gene profiles (*KIR2DL2*+/*KIR2DS2*-) are rare, they have previously been described in other Caucasian as well as North American populations [51]. A possible explanation for these rare haplotypes could be *KIR2DS2* deletion from the original cluster, or alternatively, the variant *KIR* gene profile might stem back to an ancestral haplotype existing before the duplicative event responsable for the two paralogues [57]. The presence of this variant *KIR* gene profile (*KIR2DL2*+/*KIR2DS2*-) in the group of severely ill SARS-CoV-2 patients leads to the hypothesis that the presence of *KIR2DL2* and the absence of *KIR2DS2* determine a lower efficiency of NK cell-mediated defense against viral infection which consequently increases the risk of serious clinical manifestations.

The reduction of *KIR2DS2* and *KIR2DL2* in Group S patients can also be partially correlated to the statistically significant higher frequency of the *KIR* A/A haplotype in this group of patients compared to those of Group A [34.6% vs 23.8%, OR 1.7 (95% CI 1.1–2.7); P = 0.02, Pc 0.04]. *KIR* A haplotype does not contain *KIR2DS2* and is characterized by the presence of a single activating *KIR* gene that is often non-functional (*KIR A/A KIR2DS4 DV* homozygous) and a series of inhibitory *KIR* genes with the exception of *KIR2DL2* and *KIR2DL5*.

Also the *cA01/cA01 KIR* haplotype motifs were significantly more frequent in Group S [44.2% vs 31.3%, OR 1.7 (95% CI 1.1–2.7), P = 0.01, Pc = 0.03]. These *KIR* haplotype motifs are characterized by the absence of the *KIR2DS2* activating gene and confirms the increased risk of a severe disease course in patients lacking this gene. This hypothesis is furthermore supported by the results emerging from the analysis of *KIRs* and their *HLA* ligands as well as *KIR* and *HLA* functional units (Table 5).

**Table 5:** Comparisons of *KIR* genes and their cognate ligands among COVID-19 patients divided according to severity of the clinical manifestations.

	Group A 240 patients	Group S 156 patients	P value*	OR (95% CI) A vs S	Pc^
	n (%)	n (%)			
***KIR* Ligands**					
***C1/C1***	57 (0.237)	45 (0.288)	0.29	1.3 (0.8–2.1)	0.87
***C2/C2***	63 (0.263)	48 (0.308)	0.36	1.5 (0.8–1.9)	1
***C1/C2***	120 (0.500)	63 (0.404)	0.06	0.7 (0.5–1.0)	0.18
***HLA Bw4***	183 (0.763)	123 (0.788)	0.62	1.2 (0.7–1.9)	1
**- Bw4 Ile80**	168 (0.700)	99 (0.635)	0.19	0.7 (0.5–1.1)	0.38
**-Bw4 Thr80**	57 (0.238)	36 (0.231)	0.90	1.0 (0.6–1.6)	1
**HLA *Bw6***	201 (0.838)	135 (0.865)	0.48	1.2 (0.7–2.2)	0.96
**Activating *KIR/HLA* ligands**					
***2DS1+*/*HLA C2+***	87 (0.363)	52 (0.333)	0.59	0.9 (0.6–1.3)	1
***2DS2+*/*HLA C1+***	120 (0.500)	52 (0.333)	0.001	0.5 (0.3–0.8)	0.002
*2DS4+*/HLA-A*11 or -C*04+	59 (0.246)	42 (0.269)	0.64	1.1 (0.7–1.8)	1
***3DS1+*/*HLA Bw4+***	72 (0.300)	51 (0.327)	0.58	1.1 (0.7–1.7)	1
**Inhibitory *KIR/HLA* ligands**					
***2DL1+*/*HLA C2+***	183 (0.763)	111 (0.712)	0.29	0.8 (0.5–1.2)	0.58
***2DL2+*/*HLA C1+***	120 (0.500)	57 (0.365)	0.01	0.6 (0.4–0.9)	0.02
***2DL3+*/*HLA-C1+***	162 (0.675)	105 (0.673)	1.00	1.0 (0.6–1.5)	1
***3DL1+*/*HLA-Bw4+***	171 (0.713)	117 (0.750)	0.42	1.2 (0.8–1.9)	0.84
***2DL2-3+/HLA C1+***	105 (0.438)	54 (0.346)	0.08	0.7 (0.4–1.0)	0.16

Different combinations of activating and inhibitory *KIR* genes with their ligands were analyzed and compared between patients with a-paucisymptomatic disease (Group A) and patients with severe disease (Group S).

Pc^ = P value corrected for multiple comparisons. Abbreviations: OR = odds ratio; CI = confidence interval; + = present.

Despite a substantial overlap of the *HLA* ligand frequencies in the two groups of patients, when the *KIR*/*HLA* functional units were analyzed, a reduced frequency of *KIR2DS2*/*HLA C1* and *KIR2DL2*/*HLA C1* were observed in Group S compared to Group A patients [33.3% vs 50.0%, OR = 0.5 (95% CI 0.3–0.8), P = 0.001, Pc = 0.002 and 36.5% vs 50.0%, OR = 0.6 (95% CI 0.4–0.9), P = 0.01, Pc = 0.02 respectively. However, the statistical significance found for *KIR2DL2*/*HLA C1* was lower than that found for *KIR2DS2*/*HLA C1* in the group of patients with severe clinical manifestations [60].

The possible impact of the *KIR2DS2*/*HLA C1* functional unit on the evolution of SARS-CoV-2 infection was investigated by first comparing its frequency between Group A (N = 120/240) and the population group (N = 177/400). Subsequent comparisons were made among patients stratified according to the severity of the COVID-19 clinical manifestations [group A (N = 120/240), alive in Group S (N = 50/142) and deceased patients in Group S (N = 2/14)] (Fig 1). The results showed a drop in frequency starting from 50.0% in Group A patients, through 35.2% in subjects with severe symptoms (alive in Group S) to a final low of 14.3% in people who died in intensive care units (ICU). More specifically, the frequencies of the *KIR2DS2*/*HLA C1* functional unit differed significantly between patients alive in Group S and those in Group A [35.2% vs 50.0%, OR = 0.54 (95% CI 0.35–0.85); P = 0.006]. Even between the deceased patients of Group S (despite their numbers being particularly small) and patients in Group A, the difference calculated for frequencies of the *KIR2DS2*/*HLA C1* combination reached statistical significance [14.3% vs 50.0%, OR = 0.17 (95% CI 0.02–0.78); P = 0.012].

### Multivariate analysis of clinical, immunological and genetic factors and the clinical manifestations of SARS-CoV-2 infection

Multivariate analysis based on a logistic regression model (Table 6) was used to adjust for age and gender (the most relevant factors in the comparisons between Group S and Group A). The analysis included all the clinical immunological and genetic variables found significantly associated (P value < 0.05 in univariate analysis) with the course of the viral infection: age ≥ 65 yr, gender, flu vaccine, concomitant autoimmune diseases, *KIR2DS2*, *KIR2DL2*, *KIR* haplotype AA, the *cA01/cA01 KIR* gene content motifs, the *KIR2DL2*/*HLA C1* and *KIR2DS2*/*HLA C1 KIR*-ligand combinations. Two variables, β-thalassemic trait and HLA-B*58: *01*, C*07:*01*, DRB1*03:*01* three-loci haplotype, were present in less than 10 subjects per group and were therefore excluded from the analysis.

**Table 6:** Multivariate analysis of clinical and immunogenetic factors associated with the severity of the SARS-CoV-2 disease course.

Characteristics of Sardinian COVID-19 patients	Total patients (N = 396)		Group A (N = 240)		Group S (N = 156)		Comparisons of Group S vs Group A						
							Univariate Analysis			Multivariate analysis			
	**n**	**%**	**n**	**%**	**n**	**%**	**OR**	**95% CI**	**P**_U_^*^	**OR**_**M**_^**#**^	**95% CI**_**M**_^**§**^	**P**_M_^*^	**P**_MC_^*^
Age ≥ 65 yr	120	30.3	48	20.0	72	46.2	3.4	2.1–5.5	**5.5·10**^**−8**^	5.0	3.0–8.5	**1.2·10**^**−9**^	**1.2·10**^**−8**^
Male gender	195	49.2	84	35.0	111	71.2	4.6	2.9–7.3	**1.6·10**^**−12**^	6.2	3.8–10.3	**4.1·10**^**−13**^	**4.1·10**^**−12**^
FLU vaccine 2019	52	13.1	39	16.3	13	8.3	0.5	0.2–0.9	**0.023**	0.5	0.2–0.9	**0.037**	
Autoimmune disease^&^	75	18.9	33	13.7	42	26.9	2.3	1.3–4.0	**0.002**	3.2	1.7–6.1	**4.0·10**^**−4**^	**4.0·10**^**−3**^
*KIR* haplotype AA	111	28.0	57	23.8	54	34.6	1.7	1.1–2.7	**0.02**	1.5	0.9–2.5	0.110	
*KIR2DS2+*/*HLA C1+*	172	43.4	120	50.0	52	33.3	0.5	0.3–0.8	**0.001**	0.4	0.3–0.7	**4.6·10**^**−4**^	**4.6·10**^**−3**^
*KIR2DL2+*/*HLA C1+*	177	44.7	120	50.0	57	36.5	0.6	0.4–0.9	**0.01**	0.5	0.3–0.9	**0.011**	
*KIR2DS2*	244	61.6	162	67.5	82	52.6	0.5	0.3–0.8	**0.003**	0.6	0.4–0.9	**0.03**	
*KIR2DL2*	249	62.9	162	67.5	87	55.8	0.6	0.4–0.9	**0.02**	0.7	0.4–1.2	0.170	
*cA01/cA01*	144	36.4	75	31.3	69	44.2	1.7	1.1–2.7	**0.01**	1.5	0.9–2.4	0.092	

In the comparisons between Group S and Group A, age and gender were the most relevant factors. Therefore, the odds ratios of all variables were adjusted accordingly. The Table shows the adjusted odds ratios of the variables which in univariate analysis resulted to be significantly different in the two groups of patients.

*P_U_ = P value in univariate analysis; P_M_ = P value in multivariate regression analysis. P_MC_ = P value in multivariate analysis after correction for multiple tests.

^#^OR_M_ = Odds ratio adjusted for age and gender; ^§^95% CI_M_ = 95% confidence interval calculated using the logistic regression model

^&^rheumatoid arthritis, type I diabetes mellitus and autoimmune hepatitis; + = present.

The results confirmed the strong correlation between the severe clinical manifestations of SARS-CoV-2 infection and three clinical factors: age ≥ 65 years [OR_M_ = 5.0 (95% CI 3.0–8.5), *P*_*M*_ = 1.2·10^−9^, *P*_*MC*_ = 1.2·10^−8^], male gender [OR_M_ = 6.2 (95% CI 3.8–10.3), *P*_*M*_ = 4.1·10^−13^, *P*_*MC*_ = 4.1·10^−12^] and autoimmune comorbidities [OR_M_ = 3.2 (95% CI 1.7–6.1), *P*_*M*_ = 4.0·10^−4^, *P*_*MC*_ = 4.0·10^−3^]. After Bonferroni correction, the only genetic variable that maintained considerable statistical power following multivariate analysis was represented by the *KIR*-ligand combination *KIR2DS2*/*HLA C1* [OR_M_ = 0.5 (95% CI 0.3–0.8), *P*_*M*_ = 0.0005, *P*_*MC*_ = 0.005]. This evidence indicates a strong role for this *KIR*-ligand combination in protection against severe and life-threatening disease (Table 6).

## Discussion

More than a year has passed since COVID-19 caused by SARS-CoV-2 infection was officially declared a worldwide public health concern. During this time, global and combined international research efforts have answered many of the questions concerning the disease and its modes of transmission, clinical course and risk factors. On the other hand, advances in treatment options are disappointing and despite the recent introduction of effective vaccines, an ongoing increase of mild, severe and fatal COVID-19 cases continues to be reported in most European countries, with alarming rises registered in non-Schengen areas, such as India and Brazil. The likelihood of severe illness is significantly higher in persons over 65 years of age, particularly males.

Other risk factors include hypertension, diabetes, cardiovascular disease, chronic respiratory disease, compromised immune status, cancer and obesity. The comorbidities that we found associated with an increased risk for severe disease were represented by autoimmune diseases (Hashimoto thyroiditis, rheumatoid arthritis, type I diabetes mellitus and autoimmune hepatitis). In these autoimmune disorders, it is likely that impaired cell-mediated immune response mechanisms may increase the release of proinflammatory cytokines and chemokines by T cells—leading to the so-called cytokine storm—and thus cause the life-threatening inflammatory syndromes seen in the course of COVID-19 [6]. Autoimmune diseases are a common finding in the Sardinian population, but because of the limited numbers of subjects affected by each of the aforesaid autoimmune disorders, it was not possible to distinguish their individual influence on the outcome of SARS-CoV-2 infection.

Another interesting finding was the lower frequency of severe clinical manifestations in patients with a recent history of flu vaccination, thereby confirming the partially protective effect of the flu vaccine described in previous reports [61, 62]. Also the genetically inherited β^0^39-thalassemia mutation exhibited a potentially protective effect against severe symptoms and death [59, 63]. However, the statistical power obtained for these two associations was not strong enough to draw definite conclusions and further studies on larger patient samples will be warranted to clarify these findings.

One of the topics that is currently being extensively investigated but which continues to baffle scientists worldwide is why people react so differently to being infected with SARS-CoV-2. Alongside factors that contribute to disease severity such as the environment, lifestyle and pre-existing conditions, there is a growing intensity of research into the role of the immune system. Indeed, in order to guarantee safe and appropriate care to patients affected by COVID-19, it will be important to identify markers that are able to predict how severe the disease will be and how it will progress.

Innate immune responses contribute to the control of viral replication before the onset of the more specific adaptive immune responses. Within this context, NK cells play a vital role in the eradication of viral infections, including those of the airways such as respiratory syncytial virus and influenza virus [27, 29, 32].

In the human lung, NK cells comprise up to 20% of all lymphocytes, most of which are CD56dimCD16+. Here they represent a highly differentiated population with a CD57+NKG2A− phenotype and high expression of killer-cell immunoglobulin-like receptors [36]. Surface receptors of NK cells are capable of recognizing virally infected cells as well as stressed cells, pathogens and tumors. Killer-cell immunoglobulin-like receptors are prominent mediators of NK cell function and the transition of NK cells from a quiescent state to activation largely relies on interactions between KIRs and their HLA-C Class I ligands. The impact of these interactions on immune response to SARS-CoV-2 infection has yet to be unveiled.

Preliminary studies suggest that following SARS-CoV-2 infection, a reduction in the number of circulating NK cells and/or a prevalently inhibitory receptor phenotype hint toward the dampening of NK cell responses by coronaviruses [64]. Our study is in line with these observations, highlighting that an NK cell inhibitory *KIR* gene receptor profile prevails in patients with SARS-CoV-2 infection. In particular, our patients had a higher frequency of the *KIR2DL1* and *KIR2DL3* inhibitory receptors (Table 2). It can be assumed that patients with these inhibitory *KIR* gene profiles are more susceptible to contracting SARS-CoV-2 viral infection.

The negative effect suspected for the prevalently inhibitory *KIR* gene receptor profile in the battle against SARS-CoV-2 infection became increasingly evident when patients with severe clinical manifestations (Group S) were compared to a-paucisymptomatic patients (Group A). The group of severely ill patients resulted to have a significantly higher frequency of the *KIR* A/A haplotype which contains *KIR* genes that mainly code for receptors with an inhibitory function and a single activating but often non-functional KIR2DS4 receptor (Table 3). Indeed, around 60% of these patients carried the 22bp deletion variant of the *KIR2DS4* gene which is not expressed on the NK cell surface. The frameshift mutation caused by this deletion in exon 5 translates into a truncated KIR2DS4 protein that loses the transmembrane and cytoplasmic domains of the full-length KIR2DS4 protein and can therefore only be secreted in a soluble form [52, 65].

An even more interesting finding was the significant reduction of the gene coding for the KIR2DS2 activating receptor in the group of hospitalized patients with severe forms of infection (Table 3). The *KIR2DS2* activating *KIR* gene codes for a receptor that is labeled with the CD158b monoclonal antibody and pertains to the phenotypic profile of the memory-like human lung NK cells (CD158b+/NKG2C+/CD57+/NKG2A-). Compared to conventional NK cells, this subtype of NK cells are capable of strong cytotoxic activity and effector functions and have been shown to effectively counteract a wide spectrum of human viruses (hantavirus, chikungunya virus, cytomegalovirus, and type I human immunodeficiency virus) including SARS-CoV-1 [35, 66].

The reduction observed for the *KIR2DS2* activating *KIR* gene in Group S patients acquired additional relevance in the analysis combining the *KIR2DS2* gene to its high affinity ligands of the HLA C1 ligand group (Table 4). This highly significant finding points to a protective effect of the *KIR2DS2*/HLA C1 functional unit which probably stimulates effective activity of NK cells against the virus and thereby contributes to viral clearance in the early stages of the infection. Indeed, eradication of the virus at an early stage holds the key to avoiding the severe clinical manifestations of COVID-19 such as acute lung injury and the development of acute respiratory distress syndrome (ARDS) [67].

Conversely, the *KIR* A/A haplotype and/or any *KIR* gene profile with a predominantly inhibitory effect on NK cell function, would promote ongoing viral replication and more prolonged inflammatory responses that could act as the catalyst for the significant morbidity and mortality associated with SARS-CoV-2 infection.

Market and colleagues, after analyzing reports in the literature describing NK cell phenotype and function during the SARS, MERS, and novel COVID-19 epidemics, hypothesized a dual role for NK cells in counteracting SARS-CoV-2 infection [68]. On the one hand, effective and early action of NK cells is beneficial and contributes to viral clearance and, on the other, a lower efficiency of NK cells limits response to SARS-CoV-2 infection, which is furthermore complicated by the immune evasion strategies exploited by the virus. All this results in excessive and prolonged stimulation of the immune system with the progressive accumulation of infected epithelial cells, inflammatory monocyte-macrophages and neutrophils in the lungs which, in turn, leads to the production of chemokines and cytokines and further recruitment of immune cells, including NK cells, to the lungs. Interferon-gamma (IFN-γ) is predominantly produced by lymphoid cells including T cells, NK cells and other innate lymphoid cells. Considering that NK cells are among the main producers of IFN-γ, they are possibly among the leading culprits behind the cytokine storm led by IFN-γ that triggers inflammation-mediated acute lung injury, acute respiratory distress syndrome (ARDS), systemic inflammatory response syndrome (SIRS)/sepsis and subsequent morbidity and mortality associated with COVID-19 [69, 70].

The different *KIR* gene profiles that we observed in the two groups of a-paucisymptomatic (Group A) and severely ill patients (Group S) may at least partly explain the variability in the response of NK cells and the different ways they employ to effectively counteract SARS-CoV-2 infection.

The multivariate analysis (Table 6) performed in our study, which included the most significant clinical and immunogenetic variables observed in our two groups of patients, confirmed that the strong protective effect of the *KIR2DS2*/*HLA C1* functional unit against COVID-19 was independent of all other variables including sex, age and autoimmune disease. The impact of this combination (*KIR2DS2*/*HLA C1*) on the evolution of SARS-CoV-2 became increasingly apparent when we observed the patients grouped according to disease severity (Fig 1). In fact, it had a frequency inversely proportional to the severity of the clinical manifestations, progressing from 50.0% in a-paucisymptomatic patients (group A) to 35.2% in patients with severe symptoms (alive in group S) and 14.3% in patients who died of the illness in the ICU.

The contribution of NK cell activity in early viral clearance and late immunopathology has yet to be extensively studied. In the era of COVID-19, this challenge becomes particularly important and will require the accelerated and collaborative efforts of researchers worldwide This is the first report to investigate the role of *KIR* genes and combinations of *KIR* genes and their *HLA* ligands in the pathogenesis of COVID-19. Our findings were obtained on a small and rather genetically homogeneous population in Italy and would certainly benefit from studies on larger sample sizes and/or populations with distinct genetic background and ancestry. However, the frequencies of *KIR* genes, *KIR* haplotypes and *KIR*-*HLA* ligand combinations of the Sardinian population are comparable to those observed in Northern American and other Caucasian populations [71], suggesting that our data may be valuable in determining the risks of COVID-19 in other populations. In the Sardinian population, the presence of the *KIR2DS2* gene in combination with HLA-C alleles of the C1 group seems to represent a reliable marker for the prediction of NK cell function, disease course and outcome. Combined to other clinical and immunogenetic markers, it can be used by the physician to identify patients at high risk of developing the severe clinical manifestations of COVID-19. High-risk patients could then benefit from early therapeutical intervention against SARS-CoV-2 with drugs such as remdesivir, hyperimmune plasma and/or monoclonal antibodies which are currently mainly restricted to the overt phases of the infection i.e. when the patient’s respiratory and other body functions are already compromised.

Furthermore, our findings provide a rationale for pursuing NK cell-based therapies such as CAR-NK (chimeric-antigen receptor-engineered NK) cell therapy [68] in the battle against COVID-19. Although NK cell-based therapies have mostly been developed for use against cancer, similar concepts and mechanisms could serve as a guide in the current battle against the virus. Taken together, our study only adds another small piece of knowledge toward solving the countless dilemmas surrounding the prevention and treatment of COVID-19. Larger multicenter studies in other populations as well as experimental functional studies will be needed to confirm our findings and further pursue the effect of KIR receptors on NK cell immune-mediated response to SARS-Cov-2 infection.

## References

[1] World Health Organization. (2020). Virtual press conference on COVID-19. https://www.who.int/docs/default-source/coronaviruse/transcripts/who-audio-emergencies-coronavirus-press-conference-full-and-final-11mar2020.vpdf?sfvrsn=cb432bb3_2 [Accessed April 21, 2021]

[2] WHO Solidarity Trial Consortium, Pan H, Peto R, Henao-Restrepo AM, Preziosi MP, Sathiyamoorthy V, et al. Repurposed Antiviral Drugs for Covid-19—Interim WHO Solidarity Trial Results. N Engl J Med 2020 Dec 2:NEJMoa2023184. doi: 10.1056/NEJMoa2023184 33264556 PMC7727327

[3] Melis M, Littera R. Undetected infectives in the Covid-19 pandemic. Int J Infect Dis 2021 Jan. doi: 10.1016/j.ijid.2021.01.010 33434673 PMC7837159

[4] Backer JA, Klinkenberg D, Wallinga J. Incubation period of 2019 novel coronavirus (2019-nCoV) infections among travellers from Wuhan, China, 20–28 January 2020. Euro Surveill 2020;25(5):pii = 2000062. doi: 10.2807/1560-7917.ES.2020.25.5.2000062 32046819 PMC7014672

[5] Lai CC, Shih T-P, Ko W-C, Tang H-J, Hsueh P-R. Severe acute respiratory syndrome coronavirus 2 (SARS-CoV-2) and coronavirus disease-2019 (COVID-19): The epidemic and the challenges. Int J of Antimicrob Agents 2020;55(3):105924. doi: 10.1016/j.ijantimicag.2020.105924 32081636 PMC7127800

[6] Ye Q, Wang B, Mao J. The pathogenesis and treatment of the `Cytokine Storm’ in COVID-19. J Infect 2020;80:607–13. doi: 10.1016/j.jinf.2020.03.037 32283152 PMC7194613

[7] Kochi AN, Tagliari AP, Forleo GB, Fassini GM, Tondo C. Cardiac and arrhythmic complications in patients with COVID‐19. J of Cardiovasc Electrophysiol 2020;31:1003–8. doi: 10.1111/jce.14479 32270559 PMC7262150

[8] Baig AM. Neurological manifestations in COVID‐19 caused by SARS‐CoV‐2. CNS Neurosci Ther 2020;26:499–501. doi: 10.1111/cns.13372 32266761 PMC7163592

[9] I-Cheng L, Huo T-I, Huang Y-H. Gastrointestinal and liver manifestations in patients with COVID-19. J Chin Med Assoc 2020 doi: 10.1097/JCMA.0000000000000319 32243269 PMC7176263

[10] Phelan AL, Katz R, Gostin LO. The novel coronavirus originating in Wuhan, China: challenges for global health governance. JAMA 2020;323:709–10. doi: 10.1001/jama.2020.1097 31999307

[11] Li Q, Guan X, Wu P, Wang X, Z Lei, Tong Y et al. Early transmission dynamics in Wuhan, China, of novel coronavirus‐infected pneumonia. N Engl J Med 2020;382:1199–207. doi: 10.1056/NEJMoa2001316 31995857 PMC7121484

[12] Shlomai A, Ben-Zvi H, Glusman Bendersky A, Shafran N, Goldberg E, Sklan EH. Nasopharyngeal viral load predicts hypoxemia and disease outcome in admitted COVID-19 patients. Crit Care. 2020;24:539. doi: 10.1186/s13054-020-03244-3 32873316 PMC7459243

[13] Janice Oh H-L, Ken‐En Gan S, Bertoletti A, Tan Y-J. Understanding the T cell immune response in SARS coronavirus infection. Emerg Microbes Infect 2012; 1:e23. doi: 10.1038/emi.2012.26 26038429 PMC3636424

[14] Smed-Sörensen A, Oh DY, Oshiumi H, Hsu AC. Editorial: Emerging Viruses: Host Immunity and Novel Therapeutic Interventions. Front Immunol 2018;9:2828. doi: 10.3389/fimmu.2018.02828 30555490 PMC6284039

[15] Mangalmurti N, Hunter CA. Cytokine Storms: Understanding COVID-19. Immunity 2020; 53:19–25. doi: 10.1016/j.immuni.2020.06.017 32610079 PMC7321048

[16] Kikkert M. Innate immune evasion by human respiratory RNA viruses. J Innate Immun. 2020; 12:4–20. doi: 10.1159/000503030 31610541 PMC6959104

[17] Janeway CA Jr, Medzhitov R. Innate immune recognition. Annu Rev Immunol 2002;20:197–216. doi: 10.1146/annurev.immunol.20.083001.084359 11861602

[18] McNab F, Mayer-Barber K, Sher A, Wack A, O’Garra A. Type I interferons in infectious disease. Nat Rev Immunol 2015;15:87–103. doi: 10.1038/nri3787 25614319 PMC7162685

[19] Takeuchi O, Akira S. Innate immunity to virus infection. Immunol Rev 2009;227:75–86. doi: 10.1111/j.1600-065X.2008.00737.x 19120477 PMC5489343

[20] Martin TR, Frevert CW. Innate immunity in the lungs. Proc Am Thorac Soc 2005;2:403–11. doi: 10.1513/pats.200508-090JS 16322590 PMC2713330

[21] Murira A, Lamarre A. Type-I interferon responses: from friend to foe in the battle against chronic viral infection. Front Immunol 2016;7:609. doi: 10.3389/fimmu.2016.00609 28066419 PMC5165262

[22] Paolini R, Bernardini G, Molfetta R, Santoni A. NK cells and interferons. Cytokine Growth Factor Rev 2015;26:113–20. doi: 10.1016/j.cytogfr.2014.11.003 25443799

[23] Vivier E, Raulet DH, Moretta A, Caligiuri MA, Zitvogel L, Lanier LL, et al. Innate or adaptive immunity? The example of natural killer cells. Science 2011;331:44–9. doi: 10.1126/science.1198687 21212348 PMC3089969

[24] Lodoen MB, Lanier LL. Natural killer cells as an initial defense against pathogens. Curr Opin Immunol 2006;18:391–8. doi: 10.1016/j.coi.2006.05.002 16765573 PMC7127478

[25] Brandstadter JD, Yang Y. Natural killer cell responses to viral infection. J Innate Immun 2011;3:274–9. doi: 10.1159/000324176 21411975 PMC3128146

[26] Jost S, Altfeld M. Control of human viral infections by natural killer cells. Annu Rev Immunol 2013;31:163–94. doi: 10.1146/annurev-immunol-032712-100001 23298212

[27] Li F, Zhu H, Sun R, Wei H, Tian Z. Natural killer cells are involved in acute lung immune injury caused by respiratory syncytial virus infection. J Virol 2012;86:2251–8. doi: 10.1128/JVI.06209-11 22171263 PMC3302418

[28] Frank K and Paust S. Dynamic Natural Killer Cell and T Cell Responses to Influenza Infection. Front. Cell. Infect. Microbiol 2020;10:425. doi: 10.3389/fcimb.2020.00425 32974217 PMC7461885

[29] Abdul-Careem MF, Mian MF, Yue G, Gillgrass A, Chenoweth MJ, Barra NG, et al. Critical role of natural killer cells in lung immunopathology during influenza infection in mice. J Infect Dis 2012;206:167–77. doi: 10.1093/infdis/jis340 22561366

[30] Zhou G, Juang SWW, Kane KP. NK cells exacerbate the pathology of influenza virus infection in mice. Eur J Immunol 2013;43:929–38. doi: 10.1002/eji.201242620 23436540

[31] McKinstry KK, Alam F, Flores-Malavet V, Nagy MZ, Sell S, Cooper AM, et al. Memory CD4 T cell-derived IL-2 synergizes with viral infection to exacerbate lung inflammation. PLoS Pathog 2019;15:e1007989. doi: 10.1371/journal.ppat.1007989 31412088 PMC6693742

[32] Scharenberg M, Vangeti S, Kekäläinen E, Bergman P, Al-Ameri M, Johansson N, et al. Influenza A virus infection induces hyperresponsiveness in human lung tissue-resident and peripheral blood NK cells. Front Immunol 2019;10:1116. doi: 10.3389/fimmu.2019.01116 31156653 PMC6534051

[33] Ghosh S, Nandi M, Pal S, Mukhopadhyay D, Chakraborty BC, Khatun M, et al. Natural killer cells contribute to hepatic injury and help in viral persistence during progression of hepatitis B e-antigen-negative chronic hepatitis B virus infection. Clin Microbiol Infect 2016;22:733.e9–733.e19. doi: 10.1016/j.cmi.2016.05.009 27208430

[34] Littera R, Zamboni F, Tondolo V, Fantola G, Chessa L, Orrù N, et al. Absence of activating killer immunoglobulin-like receptor genes combined with hepatitis C viral genotype is predictive of hepatocellular carcinoma. Hum Immunol. 2013;74:1288–94. doi: 10.1016/j.humimm.2013.05.007 23756163

[35] National Research Project for SARS, Beijing Group. The involvement of natural killer cells in the pathogenesis of severe acute respiratory syndrome. Am J Clin Pathol 2004;121:507–511. doi: 10.1309/WPK7-Y2XK-NF4C-BF3R 15080302 PMC7110090

[36] Marquardt N, Kekäläinen E, Chen P, Kvedaraite E, Wilson JN, Ivarsson MA, et al. Human lung natural killer cells are predominantly comprised of highly differentiated hypofunctional CD69– CD56dim cells. J Allergy Clin Immunol 2017;139:1321–30.e4. doi: 10.1016/j.jaci.2016.07.043 27670241

[37] Qin C, Zhou L, Hu Z, Zhang S, Yang S, Tao Y, et al. Dysregulation of Immune Response in Patients With Coronavirus 2019 (COVID-19) in Wuhan, China. Clin Infect Dis 2020;71:762–768. doi: 10.1093/cid/ciaa248 32161940 PMC7108125

[38] Soleimanian S, Yaghobi R. Harnessing Memory NK Cell to Protect Against COVID-19. Front Pharmacol 2020;11:1309. doi: 10.3389/fphar.2020.01309 32973527 PMC7468462

[39] Lanier LL. Up on the tightrope: natural killer cell activation and inhibition. Nat Immunol 2008; 9:495–502. doi: 10.1038/ni1581 18425106 PMC2669298

[40] Bashirova AA, Martin MP, McVicar DW, Carrington M. The killer immunoglobulin-like receptor gene cluster: tuning the genome for defense. Annu Rev Genomics Hum Genet 2006; 7:277–300. doi: 10.1146/annurev.genom.7.080505.115726 16824023

[41] Uhrberg M, Valiante NM, Shum BP, Shilling HG, Lienert-Weidenbach K, Corliss B, et al. Human diversity in killer cell inhibitory receptor genes. Immunity 1997;7:753–763. doi: 10.1016/s1074-7613(00)80394-5 9430221

[42] Cerwenka A, Lanier LL. Ligands for Natural Killer cell receptors: redundancy or specificity. Immunol Rev 2001;181:158–169. doi: 10.1034/j.1600-065x.2001.1810113.x 11513137

[43] Parham P. MHC class I molecules and KIRs in human history, healthy and survival. Nat Rev Immunol 2005;5:201–214. doi: 10.1038/nri1570 15719024

[44] Gumperz J, Litwin V, Philips JH, Lanier LL, Parham P. The Bw4 public epitope of HLA-B molecules confers reactivity with natural killer cell clones that express NKB1, a putative HLA receptor. J Exp Med 1995;181:1133–1144. doi: 10.1084/jem.181.3.1133 7532677 PMC2191933

[45] David G, Djaoud Z, Willem C, Legrand N, Rettman P, Gagne K, et al. Large spectrum of HLA-C recognition by killer Ig-like receptor (KIR)2DL2 and KIR2DL3 and restricted C1 specificity of KIR2DS2: dominant impact of KIR2DL2/KIR2DS2 on KIR2D NK cell repertoire formation. J Immunol 2013;191:4778–88. doi: 10.4049/jimmunol.1301580 24078689

[46] World Health Organization. (‎2020)‎. Clinical management of COVID-19: interim guidance, 27 May 2020. World Health Organization. https://apps.who.int/iris/handle/10665/332196. License: CC BY-NC-SA 3.0 IGO.

[47] Contu L, Arras M, Carcassi C, La Nasa G, Mulargia M. HLA structure of the Sardinian population: a haplotype study of 551 families. Tissue Antigens 1992;40:165–74. doi: 10.1111/j.1399-0039.1992.tb02041.x 1471143

[48] Jiang W, Johnson C, Jayaraman J, Simecek N, Noble J, Moffatt MF, et al. Copy number variation leads to considerable diversity for B but not A haplotypes of the human KIR genes encoding NK cell receptors. Genome Res 2012;22(10):1845–54. doi: 10.1101/gr.137976.112 22948769 PMC3460180

[49] Gómez-Lozano N, Vilches C. Genotyping of human killer-cell immunoglobulin-like receptor genes by polymerase chain reaction with sequence-specific primers: an update. Tissue Antigens. 2002;59:184–93. doi: 10.1034/j.1399-0039.2002.590302.x 12074708

[50] Vilches C, Castaño J, Gómez-Lozano N, Estefanía E. Facilitation of KIR genotyping by a PCR-SSP method that amplifies short DNA fragments. Tissue Antigens. 2007;70:415–22. doi: 10.1111/j.1399-0039.2007.00923.x 17854430

[51] Yawata M, Yawata N, Draghi M, Little AM, Partheniou F, Parham P. Roles for HLA and KIR polymorphisms in natural killer cell repertoire selection and modulation of effector function. J Exp Med 2006;203:633–645. Erratum in: J Exp Med 2006; 203:1131. doi: 10.1084/jem.20051884 16533882 PMC2118260

[52] Hsu KC, Liu X‐R, Selvakumar A, Mickelson E, O’Reilly RJ, Dupont B. Killer Ig‐like receptor haplotype analysis by gene content: Evidence for genomic diversity with a minimum of six basic framework haplotypes, each with multiple subsets. J Immunol 2002;169:5118–129. doi: 10.4049/jimmunol.169.9.5118 12391228

[53] Khakoo SI, Carrington M. KIR and disease: a model system or system of models? Immunol Rev. 2006;214:186–201. doi: 10.1111/j.1600-065X.2006.00459.x 17100885

[54] Marsh SG, Parham P, Dupont B, Geraghty DE, Trowsdale J, Middleton D, et al. Killer-cell immunoglobulin-like receptor (KIR) nomenclature report, 2002. Hum Immunol 2003;64:648–54. doi: 10.1016/s0198-8859(03)00067-3 12770798

[55] Martin AM, Kulski JK, Gaudieri S, Witt CS, Freitas EM, Trowsdale J, et al. Comparative genomic analysis, diversity and evolution of two KIR haplotypes A and B. Gene 2004;335:121–31. doi: 10.1016/j.gene.2004.03.018 15194195

[56] Pyo CW, Wang R, Vu Q, Cereb N, Yang SY, Duh FM, et al. Recombinant structures expand and contract inter and intragenic diversification at the KIR locus. BMC Genomics 20138;14:89. doi: 10.1186/1471-2164-14-89 23394822 PMC3606631

[57] Cisneros E, Moraru M, Gómez-Lozano N, Muntasell A, López-Botet M, Vilches C. Haplotype-Based Analysis of KIR-Gene Profiles in a South European Population-Distribution of Standard and Variant Haplotypes, and Identification of Novel Recombinant Structures. Front Immunol 2020;11:440. doi: 10.3389/fimmu.2020.00440 32256494 PMC7089957

[58] R Core Team. R: A Language and Environment for Statistical Computing. Vienna, Austria: R Foundation for Statistical Computing (2020). Available at http://www.R-project.org/

[59] Littera R, Campagna M, Deidda S, Angioni G, Cipri S, Melis M, et al. Human Leukocyte Antigen Complex and Other Immunogenetic and Clinical Factors Influence Susceptibility or Protection to SARS-CoV-2 Infection and Severity of the Disease Course. The Sardinian Experience. Front Immunol 2020;11:605688. doi: 10.3389/fimmu.2020.605688 33343579 PMC7746644

[60] Vierra-Green C, Roe D, Hou L, Hurley CK, Rajalingam R, Reed E, et al. Allele-level haplotype frequencies and pairwise linkage disequilibrium for 14 KIR loci in 506 European-American individuals. PLoS One 2012;7(11):e47491. doi: 10.1371/journal.pone.0047491 23139747 PMC3489906

[61] Noale M, Trevisan C, Maggi S, Antonelli Incalzi R, Pedone C, Di Bari M, et al. The Association between Influenza and Pneumococcal Vaccinations and SARS-Cov-2 Infection: Data from the EPICOVID19 Web-Based Survey. Vaccines (Basel). 2020;8:471. doi: 10.3390/vaccines8030471 32842505 PMC7565943

[62] Liu Y-M, Mahumud S-A, Chen X, Chen T-H, Liao K-S, Lo J-M. Carbohydrate-Binding Protein from the Edible Lablab Beans Effectively Blocks the Infections of Influenza Viruses and SARS-CoV-2. Cell Rep 2020;32:108016. doi: 10.1016/j.celrep.2020.108016 32755598 PMC7380208

[63] Wenzhong L, Hualan L. COVID-19: attacks the 1-beta chain of hemoglobin and captures the porphyrin to inhibit human heme metabolism. ChemRxiv (2020). doi: 10.26434/chemrxiv.11938173.v4

[64] Zheng M, Gao Y, Wang G, Song G, Liu S, Sun D, et al. Functional exhaustion of antiviral lymphocytes in COVID-19 patients. Cell Mol Immunol 2020;17:533–5. doi: 10.1038/s41423-020-0402-2 32203188 PMC7091858

[65] Middleton D, Gonzalez A, Gilmore PM. Studies on the expression of the deleted KIR2DS4*003 gene product and distribution of KIR2DS4 deleted and non‐deleted versions in different populations. Hum Immunol 2007;68:128–134. doi: 10.1016/j.humimm.2006.12.007 17321903

[66] Brillantes M, Beaulieu AM. Memory and Memory-Like NK Cell Responses to Microbial Pathogens. Front Cell Infect Microbiol 2020; 25;10:102. doi: 10.3389/fcimb.2020.00102 32269968 PMC7109401

[67] Wu C, Chen X, Cai Y, Xia J, Zhou X, Xu S, et al. Risk Factors Associated With Acute Respiratory Distress Syndrome and Death in Patients With Coronavirus Disease 2019 Pneumonia in Wuhan, China. JAMA Intern Med 2020;180:934–43. doi: 10.1001/jamainternmed.2020.0994 32167524 PMC7070509

[68] Market M, Angka L, Martel AB, Bastin D, Olanubi O, Tennakoon G, et al. Flattening the COVID-19 Curve With Natural Killer Cell Based Immunotherapies. Front Immunol 2020;11:1512. doi: 10.3389/fimmu.2020.01512 32655581 PMC7324763

[69] Forel J-M, Chiche L, Thomas G, Mancini J, Farnarier C, Cognet C, et al. Phenotype and functions of natural killer cells in critically-ill septic patients. PLoS One 2012;7:e50446. doi: 10.1371/journal.pone.0050446 23236375 PMC3516510

[70] Guo Y, Patil NK, Luan L, Bohannon JK, Sherwood ER. the biology of natural killer cells during sepsis. Immunology 2018;153:190–202. doi: 10.1111/imm.12854 29064085 PMC5765373

[71] Gonzalez-Galarza FF, McCabe A, Dos Santos EJM, Jones J, Takeshita L, Ortega-Rivera ND, et al. Allele frequency net database (AFND) 2020 update: gold-standard data classification, open access genotype data and new query tools, Nucleic Acids Research, Volume 48, Issue D1, 08 January 2020, Pages D783–D788, doi: 10.1093/nar/gkz1029 31722398 PMC7145554

